# Accelerated MRI reconstructions via variational network and feature domain learning

**DOI:** 10.1038/s41598-024-59705-0

**Published:** 2024-05-14

**Authors:** Ilias I. Giannakopoulos, Matthew J. Muckley, Jesi Kim, Matthew Breen, Patricia M. Johnson, Yvonne W. Lui, Riccardo Lattanzi

**Affiliations:** 1 Department of Radiology, The Bernard and Irene Schwartz Center for Biomedical Imaging, New York University Grossman School of Medicine, New York, NY 10016 USA; 2 Meta AI Research, New York, NY 10003 USA; 3 Department of Radiology, Center for Advanced Imaging Innovation and Research (CAI2R), New York University Grossman School of Medicine, New York, NY 10016 USA; 4 Vilcek Institute of Graduate Biomedical Sciences, New York University Grossman School of Medicine, New York, NY 10016 USA

**Keywords:** Attention, Compressed sensing, Cross-domain learning, Parallel imaging, Variational network, Preclinical research, Translational research

## Abstract

We introduce three architecture modifications to enhance the performance of the end-to-end (E2E) variational network (VarNet) for undersampled MRI reconstructions. We first implemented the Feature VarNet, which propagates information throughout the cascades of the network in an N-channel feature-space instead of a 2-channel feature-space. Then, we add an attention layer that utilizes the spatial locations of Cartesian undersampling artifacts to further improve performance. Lastly, we combined the Feature and E2E VarNets into the Feature-Image (FI) VarNet, to facilitate cross-domain learning and boost accuracy. Reconstructions were evaluated on the fastMRI dataset using standard metrics and clinical scoring by three neuroradiologists. Feature and FI VarNets outperformed the E2E VarNet for 4$\times$, 5$\times$ and 8$\times$ Cartesian undersampling in all studied metrics. FI VarNet secured second place in the public fastMRI leaderboard for 4$\times$ Cartesian undersampling, outperforming all open-source models in the leaderboard. Radiologists rated FI VarNet brain reconstructions with higher quality and sharpness than the E2E VarNet reconstructions. FI VarNet excelled in preserving anatomical details, including blood vessels, whereas E2E VarNet discarded or blurred them in some cases. The proposed FI VarNet enhances the reconstruction quality of undersampled MRI and could enable clinically acceptable reconstructions at higher acceleration factors than currently possible.

## Introduction

Rapid magnetic resonance (MR) imaging (MRI) techniques, such as parallel imaging (PI)^1–3^ and compressed sensing (CS)^4^, have significantly enhanced the cost-efficiency and expanded the range of applications for MRI. In subsequent advancements, researchers have formulated PI as a nonlinear inversion process rooted in CS principles^5–12^. These approaches leverage regularization techniques to cohesively optimize both the anatomical image and the coil sensitivity profiles. More recently, supervised deep learning (DL) has been used with PI to facilitate MRI reconstructions from highly accelerated acquisitions^13,14^. One of the first supervised DL-based MRI reconstruction methods was based on a variational network (VarNet)^13^, in which all the free regularization parameters in the CS iterative gradient descent scheme were learned from data instead of being set empirically. In particular, the regularizer used in the VarNet was the fields of experts (FoE) model^15^ and the gradient descent was unrolled yielding a deep neural network. In the more recent end-to-end VarNet (E2E VarNet)^16^, the gradient of the FoE was replaced with a UNET^17^ in each iteration of the gradient descent, resulting in improved performance^18–20^. Moreover, the E2E VarNet incorporated an additional UNET to estimate the coil sensitivity maps needed for PI from the auto calibration signal (ACS) k-space lines.

Several other approaches^21–23^ have integrated supervised DL into the image reconstruction pipeline. Among these, the Model-Based Deep Learning (MoDL) network used a convolutional neural network-based regularization prior while enforcing data-consistency through numerical optimization conjugate gradient blocks. As in the E2E VarNet, MoDL unrolls the iteration steps to yield a deep network. The densely interconnected network (DIRCN)^24^ adapted the E2E VarNet using input level dense connections to improve gradient and information flow as in^25,26^. DIRCN also used long range skip-connections to directly connect the UNETs in each gradient descent step. Recurrent VarNet^27^ is another adaptation of the E2E VarNet, which replaces the traditionally used UNET with a recurrent unit. In this approach a hidden state is provided as an additional input to each gradient descent step, that stores the sequence of information from the previous steps. However, the use of recurrent networks increases the memory demand of the network due to the need of accumulating more gradients in the memory. Methods like the Deep J-SENSE^28^ and Joint-ICNet^29^ also follow the unrolled optimization scheme of VarNet but refine both the image and the coil sensitivity maps through an alternating optimization model, which enables the use of a small number of ACS lines to estimate accurate coil sensitivity maps. Learned DC^30^ learns the data likelihood model in a dynamic MRI setting to better approximate the noise distribution in k-space. CTFNet^31^ can exploit spatiotemporal correlations simultaneously from both the frequency and the time domain. Additionally, studies on dynamic MR imaging such as CINENet^32^ and L+S-Net^33^ are able to operate with complex-valued data directly, avoiding potential information loss associated with treating real and imaginary components in separate channels. Although networks using complex-valued data can reduce the number of parameters and accelerate convergence, they require complex arithmetic, which can lead to greater computational and memory demands, making such models more challenging to develop and optimize. More recently, score-based approaches and self-supervised or unsupervised DL methods^34–37^ have been introduced. For example, SURE-Score^38^ combines a denoiser and a score function using only noisy training data, offering a cost-effective alternative to supervised DL-based methods. Self-Score^39^ introduced a fully-sampled-data-free score-based diffusion model that learns the MR image prior in a self-supervised manner using Bayesian deep learning.

Cross-domain learning methods, such as the KIKI-net^40^, incorporate learning in both the image-space and k-space to improved MR image reconstruction. Another example of cross-domain learning is provide by^41^, where dynamic image reconstruction is performed by iterating across the frequency-time domain and the image domain. However, such framework does not utilize an unrolled gradient descent scheme, so it does not directly preserve the physics of parallel imaging. DCT-net^42^ and the method presented in^43^ also perform cross-domain learning. In particular, DCT-net reconstructs both the image and the undersampled k-space with two networks running in parallel joined with transformer blocks. To our knowledge, both DCT-net and the method in in^43^ have not been applied yet to multi-coil parallel imaging reconstructions. DIIK-Net^44^ interleaves the image and the k-space into a cross-domain interaction block in each refinement module, thus performing cross-domain learning in each gradient descent step. DIIK-Net reports a slightly lower PSNR score than the XPDNet model^45^, which ranked below the E2E VarNet in the fastMRI public leaderboard^19^. Finally, IKWI-net^46^ performs learning using image, k-space, and a wavelet domain. However, this approach relies on magnitude DICOM images to simulate fully-sampled raw data, which leads to unrealistic results^47,48^.

DL-approaches for image reconstruction are nowadays incorporated in most commercial products. At the time of writing, self-supervised methods achieve competitive reconstruction performance compared to supervised methods, although the latter still maintain an edge. Notably, the E2E VarNet model and its extension, the DIRCN model, have secured the third and second positions, respectively, on the fastMRI leaderboard for 4$\times$ accelerated reconstructions (Supporting Fig. S1). In the case of 8$\times$ accelerations, the E2E VarNet dropped to the fourth position, whereas the Iterative Refinement with Fourier-Based Restormer reached third place^49^, and DIRCN maintained second place (Supporting Fig. S2). The reported results show that there is room for improvement to improve image reconstruction for large undersampling factors.

The aim of this work was to improve the original E2E VarNet by implementing and evaluating three modifications. First, we adapted the network’s architecture to perform training in a feature-space instead of image-space, which preserved high-level features between the iterations of gradient descent. In our approach, the feature-space data-consistency term decodes the feature space to k-space, performs data consistency, and finally encodes back to feature space. Second, we leveraged the feature-space representation of the MR image and employed a transformer^50,51^. In particular our attention mechanism in the transformer utilizes the knowledge of what aliasing artifacts look like in the case of Cartesian undersampling and attenuates them in the reconstructed images. Finally, we combined our proposed feature-space approach with the image-space representation of the E2E VarNet to build a Feature-Image (FI) VarNet, in an attempt to boost performance, by merging a comprehensive CNN model (E2E VarNet) and a CNN model augmented with attention mechanisms (Feature VarNet). The models were compared and the best one was evaluated by three neuroradiologists. A preliminary version of this work was presented at the 2023 Annual Meeting of the International Society for Magnetic Resonance in Medicine^52,53^.

## Methods

### MR image reconstruction

The MR signal $\textbf{k}_{i}$ received by the *i*-th coil is related to the MR image $\textbf{x}$ by the forward problem:

1 $$\begin{aligned} \textbf{k}_{i} = \textbf{m} \odot \textbf{F} \left( \textbf{c}_{i} \odot \textbf{x} \right) , \quad i = 1, \ldots , N. \end{aligned}$$

Here, *N* is the number of receive coils, $\textbf{c}_{i}$ are the receive coil sensitivity profiles, $\textbf{F}$ is the discretized Fourier transform operating on a vector with multichannel images concatenated, and $\textbf{m}$ is the predefined undersampling mask.

One can solve for $\textbf{x}$ by precomputing $\textbf{c}$ and inverting (1) through a regularized optimization routine based on CS^4,54^. In particular, we can express the optimization problem as:

2 $$\begin{aligned} \tilde{\textbf{x}} = \mathop {\textrm{argmin}}\limits _{\textbf{x}} \frac{1}{2} \sum \limits _{i}^{N} \left\Vert \textbf{m} \odot \textbf{F} \left( \textbf{c}_{i} \odot \textbf{x} \right) - \tilde{\textbf{k}}_{i}\right\Vert + \lambda \varvec{\mathcal {Q}}\left\{ \textbf{x}\right\} , \end{aligned}$$

where $\varvec{\mathcal {Q}}$ is the regularizer, $\lambda$ is its weighting factor, and $\tilde{\textbf{k}}_{i} = \textbf{m} \odot \textbf{k}_{i}$ is the undersampled measured k-space (signal) data. The solution of (2) for a fully sampled k-space and $\lambda = 0$ is the inverse Fourier transform. If $\varvec{\mathcal {Q}}$ is differentiable, the inverse problem can be solved with a few gradient descent iteration steps as:

3a $$\begin{aligned} \textbf{k}^{j+1}= & {} \textbf{k}^{j} - \eta ^{j} \textbf{m} \left( \textbf{k}^{j}-\tilde{\textbf{k}} \right) + \lambda \textbf{F}\mathcal {E}\frac{\partial \varvec{\mathcal {Q}}\left( \mathcal {R}\left( \textbf{F}^{-1} \textbf{k}^{j}\right) \right) }{\partial {\textbf{k}}}. \end{aligned}$$

3b $$\begin{aligned} \mathcal {E}\left\{ z\right\}= & {} \left[ \textbf{c}_1 \odot z, \dots , \textbf{c}_N \odot z \right] \end{aligned}$$

3c $$\begin{aligned} \mathcal {R}\left\{ z_1, \dots , z_N \right\}= & {} \sum \limits _{i=1}^N \textbf{c}^*_i \odot z_i \end{aligned}$$

Here, $F$, is the discretized Fourier transform operating on a vector with single channel images and $\textbf{k}$ is the vector containing the k-space from all individual coils. $\mathcal {E}$ is the expand operator, which performs the multiplication of the individual coil images with $\textbf{c}_{i}$. $\mathcal {R}$ is the reduce operator, which multiplies element-wise the conjugate of $\textbf{c}_{i}$ with the coil images and sums over the number of the coil channels. $\eta ^{j}$ is the learning rate of the gradient descent and *j* is the iteration number. Finally, the individual coil images are given from $\textbf{x}_{i} = |\textbf{F}^{-1}\textbf{k}_{i}|$ and the coil combined image is the root-sum of squares of the individual coil images.

### Variational network

The above described inverse problem remains inherently ill-posed for high undersampling rates^6^. This happens because the regularization techniques normally employed in CS rely on hand-crafted parameters that may not be suitable to reconstruct the complex details of the image^55^. In addition, a poor choice of these priors might result in excessively smooth images, or under-regularized noisy images. Motivated by these limitations, the VarNet^13^ embedded CS into a deep learning framework, where the gradient of the regularizer of (2) is learned from data, resulting in a physics-based reconstruction network. In particular, in the VarNet, $\varvec{\mathcal {Q}}^{j}$ is a FoE model for the *j*-th gradient descent iteration^15^ (a generalization of total variation) where all its parameters, including $\lambda$ are learned from data. The network is trained using an unrolled gradient descent scheme^26,56–58^ where the neural network weights in $\varvec{\mathcal {Q}}^{j}$ are updated at each step. In the original VarNet, the coil sensitivities $\textbf{c}$ are computed with the ESPIRiT method^9^ and passed as an additional input to the network along with the undersampled k-space.

The E2E VarNet^16^ addressed the limited expressive power of the FoE by substituting the gradients of $\varvec{\mathcal {Q}}^{j}$ with a UNET^17^, due to the UNET’s capacity to learn complex representations and their capability to model objects at different scales. In addition, $\textbf{c}$ is also learned in parallel with the gradient of $\varvec{\mathcal {Q}}^{j}$ during the training process of the E2E VarNet. This is done with an additional UNET that takes the low-resolution image generated using the ACS lines of k-space as an input and outputs the sensitivity maps. Each of the $J_\mathrm{ima}$ unrolled gradient descent steps (cascade^25^) of E2E VarNet is

4 $$\begin{aligned} \textbf{k}^{j+1} = \textbf{k}^{j} - \eta ^{j} \textbf{m}\odot \left( \textbf{k}^{j} - \tilde{\textbf{k}}\right) + \textbf{F} \mathcal {E}\left\{ \varvec{\mathcal {N}}^{j}\left( \mathcal {R}\left\{ \textbf{F}^{-1} \textbf{k}^{j} \right\} \right) \right\} , \end{aligned}$$

where $\tilde{k}$ is the undersampled measured k-space signal concatenated as a vector for all coil channels, $\varvec{\mathcal {N}}^{j}$ is a convolutional neural network and $j = 1, \ldots , J_\mathrm{ima}$ with $J_\mathrm{ima}$ being the number of the gradient descent steps. Individual coil images are reconstructed using the inverse Fourier transform from the fully-sampled k-space obtained at the last gradient descend step and combined using the root sum of squares to obtain the final image. The parameters of all $\varvec{\mathcal {N}}^{j}$, the learning rate of the gradient descent $\eta ^{j}$ and the sensitivities $\textbf{c}$ are learned by minimizing a cost-function between the combined image and the ground-truth image $\hat{\textbf{x}}$. The metric used in the cost-function can be any metric of choice, such as the mean and normalized mean squared error (MSE, NMSE), the peak signal-to-noise ratio (PSNR)^59^, or the structural similarity index measure (SSIM)^60^, among others.

### VarNet architecture modifications

#### Feature-space encoding

**Figure 1:**
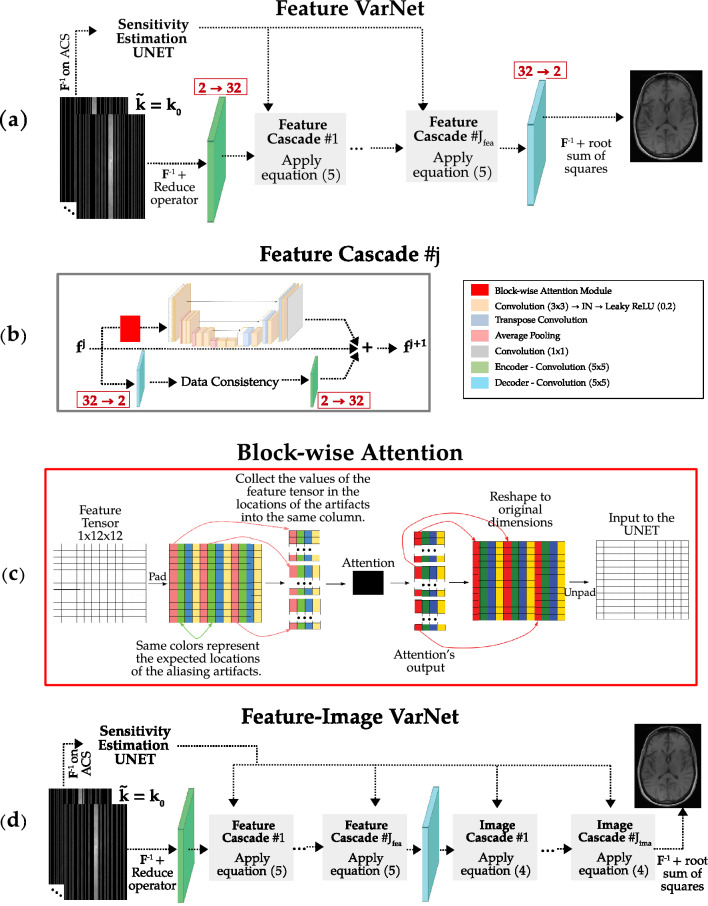
Neural network architectures. In Feature VarNet (**a**), the coil sensitivities are estimated as in the E2E VarNet with a UNET and are passed to the Feature Cascades (**b**). The k-space is encoded in feature-space, and the resulting feature maps are processed using the update rule of (5) in each cascade ($j = 1, \dots J_\mathrm{fea}$). The attention module (red square block) precedes the neural network in the Feature VarNet cascades. The attention module is incorporated by first reshaping the feature maps (tensors) to blocks, then applying attention, and finally reshaping the output feature maps back to their original dimensions, before they are processed by the neural network in the cascade. A representative example of our reshaping approach is shown in (**c**) for a feature tensor with 1 channel and width and height equal to 12. The acceleration factor is equal to 4. Due to Cartesian undersampling, the aliasing artifacts appear at regular intervals of $N = W/R = 3$ pixels. In the Feature VarNet, the output feature map of the last cascade is decoded into k-space, which is then inverse Fourier transformed to obtain the individual coil images. In the FI VarNet architecture (**d**), the feature map of the last cascade is decoded into k-space, which is further processed by the update rule of (4) in the $j = 1, \dots J_\mathrm{ima}$ image cascades. The output k-space of the last image cascade is inverse Fourier transformed to obtain the individual coil images. For both networks, the final reconstructed image is obtained as the root sum of squares combination of the individual coils images. Both Feature and FI VarNets are trained end-to-end.

In the E2E VarNet architecture and other unrolled optimization-based models^61^, most of the high-level features are discarded in the last convolutional layers of each cascade to obtain the update of the image or k-space in order to perform data consistency (4). In particular, the number of $\varvec{\mathcal {N}}^{j}$ output channels decreases from a high number (usually set to 32) to 2 (to represent the real and imaginary part of the image-space update). Nevertheless, the remaining features (30) could contain useful information for the reconstruction. Here, we propose a different approach, dubbed Feature VarNet, where we use the unrolled gradient descent algorithm as in the E2E VarNet (4), but we perform the updates of the gradient descent in a feature-space ($\textbf{f}$), instead of the k-space ($\textbf{k}$), or image-space. This approach allows us to maintain a high number of feature channels across the network’s cascades. In particular, we introduce an encoder ($\mathcal {A}$) neural network that maps $\textbf{k}$ to $\textbf{f}$, and a decoder ($\mathcal {B}$) neural network that maps $\textbf{f}$ to $\textbf{k}$. By substituting $f = \mathcal {A}\left( \textbf{k}\right)$ and $k = \mathcal {B}\left( \textbf{f}\right)$, the gradient descent step of (4) can be updated as:

5 $$\begin{aligned} \textbf{f}^{j+1} = \textbf{f}^{j} - \eta ^{j} \mathcal {A}\left( \mathcal {R}\left\{ \textbf{F}^{-1} \textbf{m} \odot \left( \textbf{F}\mathcal {E}\left\{ \mathcal {B}\left( \textbf{f}^{j}\right) \right\} - \tilde{\textbf{k}} \right) \right\} \right) - \varvec{\mathcal {N}}^{j}\left( \textbf{f}^j\right) , \end{aligned}$$

where $j = 1, \ldots , J_\mathrm{fea}$ with $J_\mathrm{fea}$ being the number of the gradient descent steps (Fig. 1 top). In this feature-space representation, each $\varvec{\mathcal {N}}^{j}$ network produces directly a feature tensor with a high number of channels (32), $\mathcal {B}$ decodes it to k-space, and data consistency is performed. The updated k-space in encoded to a feature tensor (32 channels) with $\mathcal {A}$ and is passed to $\varvec{\mathcal {N}}^{j+1}$. As a result, the tensor maintains its high number of channels throughout all $\varvec{\mathcal {N}}$, avoiding the information bottleneck that happens when the channels are reduced to 2.

We can either enforce consistency by using the same encoder and decoder to and from feature-space throughout all cascades or use independent encoder and decoder in the network. For the most part of this work we used consistent encoder and decoders, except in (Model Ablations) where we experimented with different encoder and decoders. We used single convolutional layers with kernel size of 5 and padding equal to 2 to represent the encoder and the decoder. Specifically, the encoder mapped the 2 input channels, corresponding to the real and imaginary part of the image, to a predefined number of feature channels $q=32$, and the decoder mapped back from $q=32$ to 2 channels. Both the encoder and decoder were used without an activation function. The parameters of all $\varvec{\mathcal {N}}^{j}$, the encoder, and the decoder were learned from the training data.

#### Block-wise attention

In the proposed Feature VarNet, all $\varvec{\mathcal {N}}^{j}$ can be UNETs as in the E2E VarNet. In this work we propose to precede each UNET with a self-attention layer^51^. First, we added a positional encoding to the input features to provide spatial information to the attention mechanism. Next, we modified the input using dilated convolutions to compute the query, key, and value embeddings in order to calculate the attention weights. The attention weights are then used to attend to the value embeddings and produce the output features. Finally, the output features are projected back onto the same shape as the original input features using a convolution with a $1 \times 1$ kernel, and the two are added together to produce the final output.

The matrix multiplications in the attention mechanism were performed in blocks to reduce computational complexity. In particular, we reshaped the feature tensor into a block-based representation to help the model identify the spatial location of the aliasing artifacts caused by the Cartesian undersampling. For example, consider a feature tensor of dimensions $C \times H \times W$ (channels, height, width), and acceleration rate *R*. First, we collect the elements of the tensor that are $N = W/R$ elements apart in the width into tall matrices of size $C\times (H \cdot N)$, since the aliasing artifacts appear at regular intervals of *N* voxels along the phase-encoding direction. In case the width is not divisible with the acceleration, the tensor can be padded before the reshape. After this, the resulting tall matrices are concatenated and form a feature tensor of dimensions $(H \cdot N) \times C \times R$, and a batch matrix multiplication follows^51^. This block representation helps the model identify the spatial location of the aliasing artifacts. Note that *N* and *R* adapt depending the acceleration factor. The reshaping process is depicted in Fig. 1 (bottom) for a toy example with $C \times H \times W = 1 \times 12 \times 12$ and $R=4$.

#### Feature-image variational network

Given the superior performance demonstrated by cross-domain convolutional neural networks^62^, such as the KIKI-net^40^, we combined the Feature VarNet (with attention) and the E2E VarNet into a single network. The new network, dubbed FI VarNet, combines feature-space and image-space based reconstructions to improve performance.

Figure 1 (middle) presents the FI VarNet architecture. First, the coil sensitivity maps are estimated as in the E2E and Feature VarNet approaches, and the $J_\mathrm{fea}$ gradient descent steps of equation (5) are performed (feature cascades). The resulting feature-space representation $\textbf{f}^{J_\mathrm{fea}}$ is then decoded into a k-space representation, which is passed as the initial value $\textbf{k}^{1}$ to (4). Equation (4) is solved for $J_\mathrm{ima}$ gradient descent steps (image cascades) and the final image is reconstructed. We dubbed the E2E VarNet’s cascades as image cascades for simplicity, as they refer to operations that bridge both k-space and image-space.

### Model training

#### Datasets

The datasets used in the current study were obtained from the fastMRI public database (fastmri.med.nyu.edu)^18,63^. The fastMRI dataset includes both the raw k-space data and ground-truth MRI images presented in this work. We combined the training (4469 volumes) and validation (1378 volumes) brain fastMRI datasets for training. We used the validation brain fastMRI dataset for validation. For the performance assessment (Performance Assessment), all models were tested on the entire brain fastMRI test dataset, which consisted from 558 volumes. 49 of these volumes were scanned with fluid attenuated inversion recovery (FLAIR), 187 were T1 and T1 post contrast, and 322 were T2-weighted. These ratios reflect the contrast distribution of the validation and train datasets. For the comparative study with the public fastMRI leaderboard (Leaderboard Comparison), a subset of the brain fastMRI test dataset (standard leaderboard test dataset) was used (281 volumes to evaluate 4$\times$ acceleration and 277 volumes to evaluate 8$\times$ acceleration). The clinical study (Clinical Evaluation) was implemented using a subset of the leaderboard test dataset as in^64^, consisting of 20 cases (4 FLAIR, 5 T1-weighted, and 11 T2-weighted volumes) with abnormalities (clinical dataset). The abnormalities included postsurgical complications, vascular-related conditions, masses and tumors, and fluid-related conditions. We also used the knee fastMRI dataset to determine the generalizability of our models. Since the ground-truth images for the knee fastMRI testing dataset are not publicly available, we used the knee fastMRI validation dataset (199 volumes) for testing. We used the knee fastMRI training dataset (973 volumes) for training. We skipped the validation process for the knee and applied the same hyperparameters for training that were previously used for the brain (see “Optimization and network configurations”).

#### Undersampling

We used Cartesian undersampling. We used either $8\%$, $7\%$, or $4\%$ of the central k-space as ACS lines and uniformly sampled the rest of k-space to achieve an acceleration factor (*R*) of 4, 5, or 8, respectively. All models were trained and tested using the same undersampling mask.

#### Optimization and network configurations

The optimization model used to train the neural networks in this study was based on a combination of the AdamW optimizer with a learning rate of 0.0003^65^ and a learning rate scheduler, to adjust the learning rate during training. We trained the networks using 210k iteration steps and used a custom step function for the learning rate scheduler. In particular, the step function gradually increased the learning rate from 0 to 0.0003 over a period of 7.5k steps, after which the learning rate remained constant for 140k steps. For the remaining steps, the learning rate switched to a cosine annealing schedule. The cosine annealing schedule was used to gradually decrease the learning rate from its maximum value down to a small value of $10^{-8}$, ensuring good convergence without oscillations.

The UNETs, serving as the backbone for both the E2E VarNet and the proposed VarNet models, shared a similar architecture. Specifically, we employed four layers of average pooling and transpose convolutions, each with a kernel size, stride, and padding of 2, 2, and 0, respectively. The convolution layers used a kernel size of 3 with both padding and stride set to 1. Leaky ReLU activation functions were used with a negative slope of 0.2. The complex-valued k-space data were split into a two-channel real-valued representation. The input tensors to the U-Nets, whether representing k-space data or features, were normalized to ensure that each channel had a mean of 0 and a standard deviation of 1. Finally, all networks were trained with a batch size of 1.

#### Model size

In this study, we compared the original E2E VarNet against the three proposed variations: Feature VarNet, Feature VarNet with attention, and FI VarNet. Despite differences in their architectures, for a fair comparison, all models were designed to have a similar number of parameters. In particular, we used 12 cascades and 32 feature channels in the UNETs for the E2E and Feature VarNets for all studied cases. In this setting the E2E VarNet required 93.6 million parameters, while Feature VarNet required an additional 0.3 million parameters for its encoder, decoder, and convolutions in the attention layer. Finally, FI VarNet required 93.8 million parameters for a 6 feature-6 image cascade architecture and 187 million parameters for a 12 feature-12 image cascade architecture.

### Evaluation strategy

#### Quantitative evaluation

To assess the performance of all VarNet models, we conducted a quantitative evaluation using three metrics: SSIM, PSNR, and NMSE. All models were trained on 2D input-output pairs representing individual slices of the training set’s volumes. The metrics were measured by comparing the entire 3D reconstructed volume of the sample with the corresponding ground truth and computing the volume average over the entire dataset. During training, 1-SSIM was used as the loss function to optimize the network parameters, ranging between 0 and 1, with lower values indicating better similarity. SSIM was also used as the primary evaluation metric due to its ability to capture both structural and perceptual similarities between the predicted and ground truth volumes^66^. PSNR and NMSE were also computed to provide additional quantitative measures of performance.

#### Clinical evaluation

Three neuroradiologists with 23 (Reader C), 4 (Reader B), and 2 (Reader A) years of clinical experience assessed the image quality of FI VarNet, which was found to be the best model in the quantitative evaluation among the three proposed approaches (see “Leaderboard comparison”). Each radiologist was assigned the ground-truth fully-sampled image and two undersampled reconstructions: one with the FI VarNet (12 feature-12 image cascades) and one with the pretrained E2E VarNet model^16^. All radiologists had knowledge of the ground-truth, but were blinded to the particular reconstruction model and performed their reviews independently. The readers were tasked to label the images as “FI VarNet”, “E2E VarNet”, or “cannot tell”, based on their overall quality. Additionally, three Likert-like grading scales were used to assess artifacts, sharpness, and contrast-to-noise ratio (CNR), in comparison to the ground-truth, similar to what was done in a previous study^67^. For the artifacts scale, a score of 1 indicated no artifacts present, while 2 indicated minimum artifacts that do not affect diagnostic quality. In the sharpness scale, a score of 1 indicated that the sharpness for structures and findings matched the ground-truth, while 2 indicated differences. Lastly, in the CNR scale, a score of 1 indicated equal conspicuity for structures and findings as the ground-truth, while 2 indicated differences.

## Results

All models were trained on a high-performance cluster using four NVIDIA A100 Tensor Core GPUs, each equipped with 80 GB of memory.

### Performance assessment

Table 1 (top) compares the average SSIM, PSNR, and NMSE between the E2E VarNet, Feature VarNet with and without attention, and FI VarNet. FI VarNet used 6 feature and 6 image cascades to ensure that differences in performance with respect to the Feature VarNet (12 cascades) and the E2E VarNet (12 cascades) were due to their architectural variations rather than their sizes. Feature VarNet outperformed the E2E VarNet in SSIM by 0.0002 and 0.0007 for $4 \times$ and $5 \times$ acceleration, respectively, due to the preservation of high-level features in each cascade. When the block-wise attention was incorporated the SSIM improvement increased to 0.0004 and 0.0009 for four and five-fold acceleration, respectively. The FI VarNet also outperformed the E2E VarNet in SSIM by 0.0009 and 0.0011 for four and five-fold acceleration, respectively, showing the superiority of cross-domain convolutional neural networks. Finally, the results are statistically significant as indicated by a paired t-test^68^ at a $5\%$ significant level.

**Figure 2:**
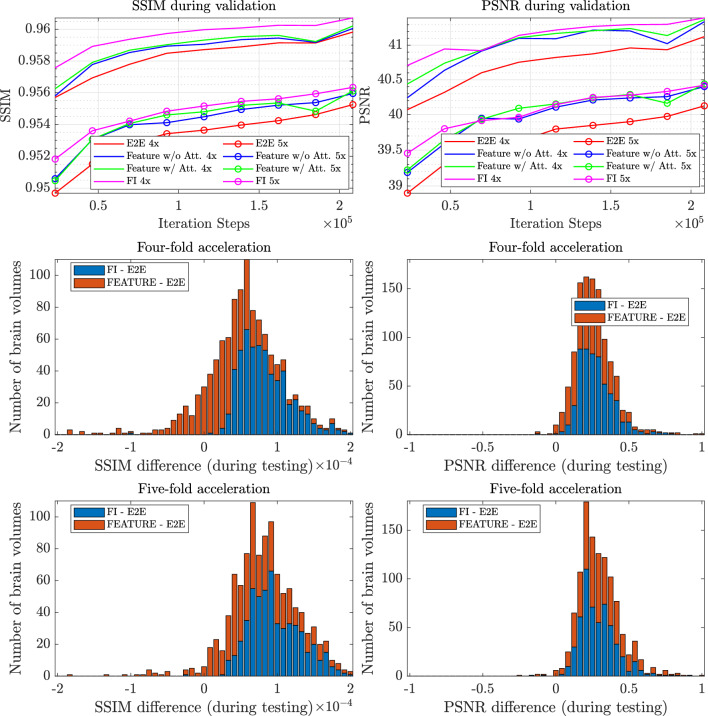
(top) Error during validation for the SSIM (left) and PSNR (right) for the FI, Feature (with and without attention) and E2E VarNets with respect to the ground-truth. The error is averaged for all brain volumes in the validation dataset. (middle and bottom) Histograms of differences in SSIM (left) and PSNR (right) for the FI VarNet and the Feature (with attention) VarNet with respect to the E2E VarNet for all brain volumes in the test dataset.

**Table 1:** Average SSIM, PSNR, and NMSE comparison on all test data for brain scans using the E2E, Feature, and FI VarNet architectures and four and five fold accelerations.

Anatomy	Network	Attention	Space	Cascades	SSIM		PSNR		NMSE ($10^{-3}$)	
					$R = 4$	$R = 5$	$R = 4$	$R = 5$	$R = 4$	$R = 5$
Brain	FI VarNet	Yes	Feature and Image	6+6	${\textbf {0.9596}}$	${\textbf {0.9552}}$	${\textbf {41.45}}$	40.46	${\textbf {3.52}}$	4.32
	Feature VarNet	Yes	Feature	12	0.9591	0.9550	41.42	${\textbf {40.48}}$	3.55	${\textbf {4.30}}$
	Feature VarNet	No	Feature	12	0.9589	0.9548	41.39	40.45	3.56	4.33
	E2E VarNet	No	Image	12	0.9587	0.9541	41.17	40.18	3.71	4.57
Knee	FI VarNet	Yes	Feature and Image	12+12	${\textbf {0.9236}}$	–	${\textbf {40.08}}$	–	${\textbf {5.17}}$	–
	E2E VarNet	No	Image	12	0.9187	–	39.93	–	5.30	–

Average errors are also shown for knee scans at four fold acceleration and the E2E and FI VarNets.

Bold values indicate the best performing model.

Figure 2 (left) presents the convergence of the validation error for PSNR and SSIM for both $4 \times$ and $5 \times$ acceleration factors. The large gain towards the end of the training is due to the cosine annealing in the optimization process. FI VarNet always maintains smaller errors than all other models in both accelerations for the SSIM. In the case of PSNR, FI is similar to Feature VarNet (with and without attention) during training and marginally outperforms them towards the end of the training, except in the case of PSNR and $5 \times$ acceleration. The Feature-based models outperform E2E VarNet in all cases during training.

Figure 2 (middle four-fold acceleration, bottom five-fold acceleration) compares the SSIM and PSNR score differences of the FI VarNet and Feature VarNet (with attention) with respect to the E2E VarNet for the entire testing dataset. Both the Feature VarNet and the FI VarNet obtained larger PSNR values than the E2E VarNet for all testing cases (except a few outliers). The FI VarNet had higher SSIM values than the E2E VarNet for almost the entire testing dataset, while the Feature VarNet had lower SSIM scores than E2E VarNet for a few cases. The latter can be attributed to their training on the SSIM metric, as both models were explicitly optimized to excel in SSIM performance. The E2E VarNet’s slightly higher SSIM scores than the Feature VarNet’s in a few cases may be due to potential overfitting to specific patterns in the training data, leading to improved SSIM performance on some examples but reduced PSNR performance.

**Figure 3:**
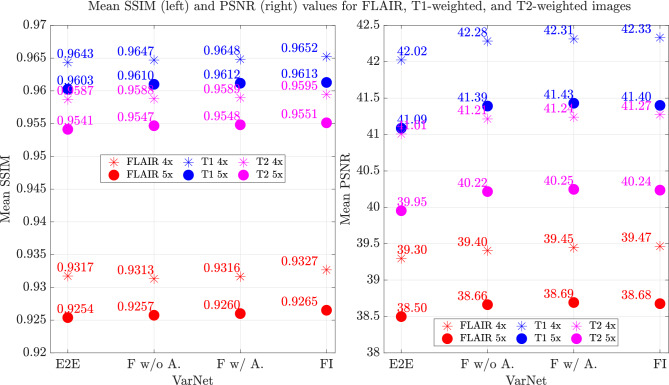
Performance of the E2E, Feature (with and without attention), and FI VarNet (x axis) for the three different contrasts (FLAIR, T1-weighted, and T2-weighted) in the brain fastMRI test dataset. The average SSIM (y axis, left) and average PSNR (y axis, right) are presented for four-fold and five-fold accelerations.

Figure 3 compares the performance of the E2E VarNet, Feature VarNet (with and without attention), and FI VarNet for the three difference image contrasts in the fastMRI test dataset and for both four-fold and five-fold accelerated reconstructions. The large average SSIM and PSNR performance for both the T1-weighted and T2-weighted image datasets was expected as the network was trained mostly on these types of contrasts. The results for the individual contrasts are in a good agreement with the average results from all contrast-weighted images, except for FLAIR images with 4x acceleration, where the E2E VarNet outperformed the Feature VarNet (w and w/o attention) in terms of SSIM. This can explain the higher SSIM scores of E2E VarNet over Feature VarNet that were observed for a few cases in the top-left histogram of Fig. 2.

**Figure 4:**
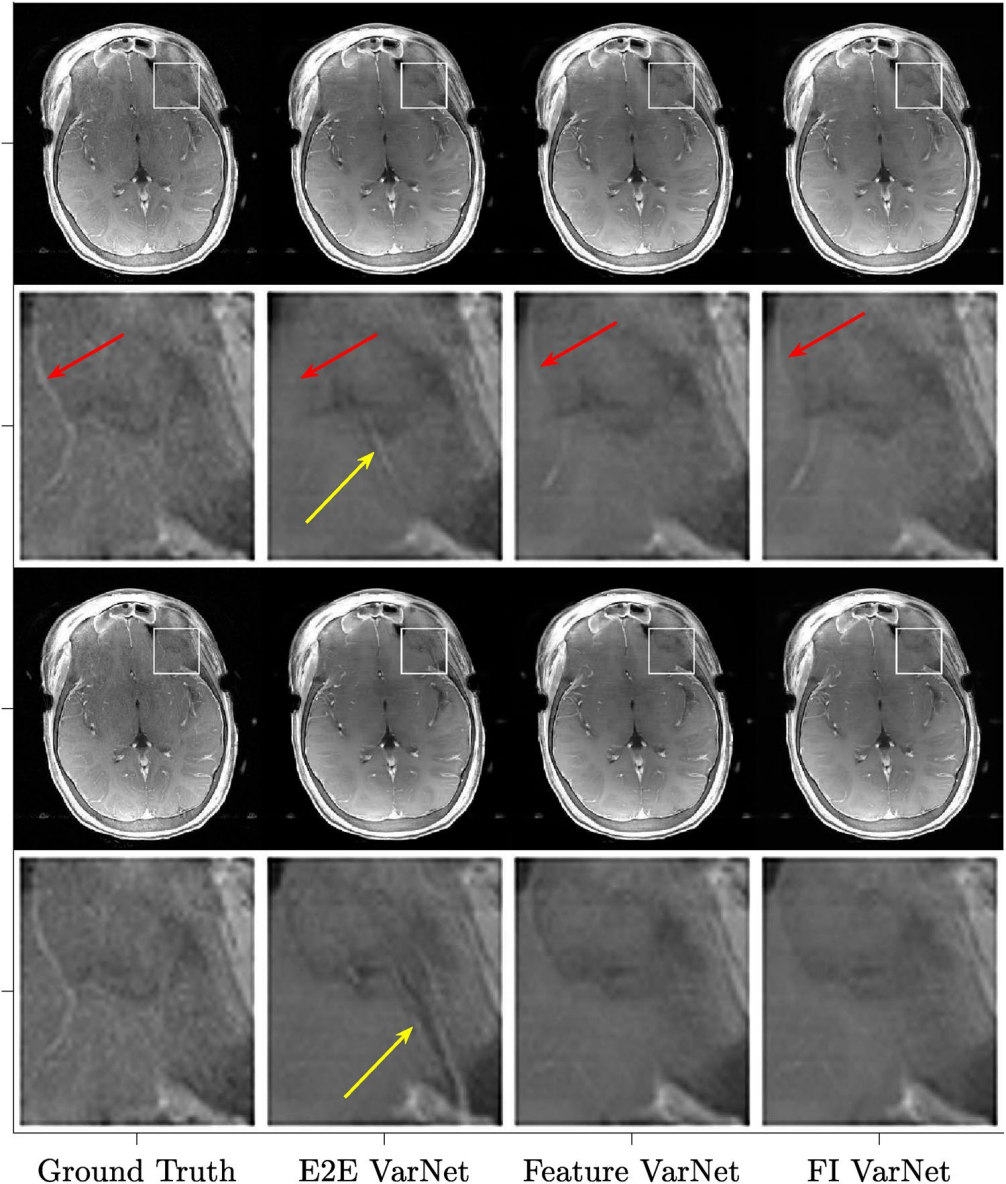
Comparison of representative image reconstructions at four-(top) and five-(bottom) fold accelerations using different VarNet architectures with matching train-test conditions. The fully-sampled ground truth reconstructions are shown on the left column. A zoomed section of interest, indicated by the white bounding box, is shown at even rows. The E2E VarNet exhibits an artifact in the zoomed area (yellow arrow), which varies with the two different acceleration factors. The artifact is absent in the Feature VarNet and the FI VarNet reconstructions. In addition, at four-fold acceleration, E2E VarNet causes blurring of the blood vessel (clearly visible on the ground-truth image), while neither the Feature VarNet nor the FI VarNet reconstructions suffer from the same artifact (red arrow). At five-fold acceleration, all networks cause a similar smoothing of the blood vessel.

**Figure 5:**
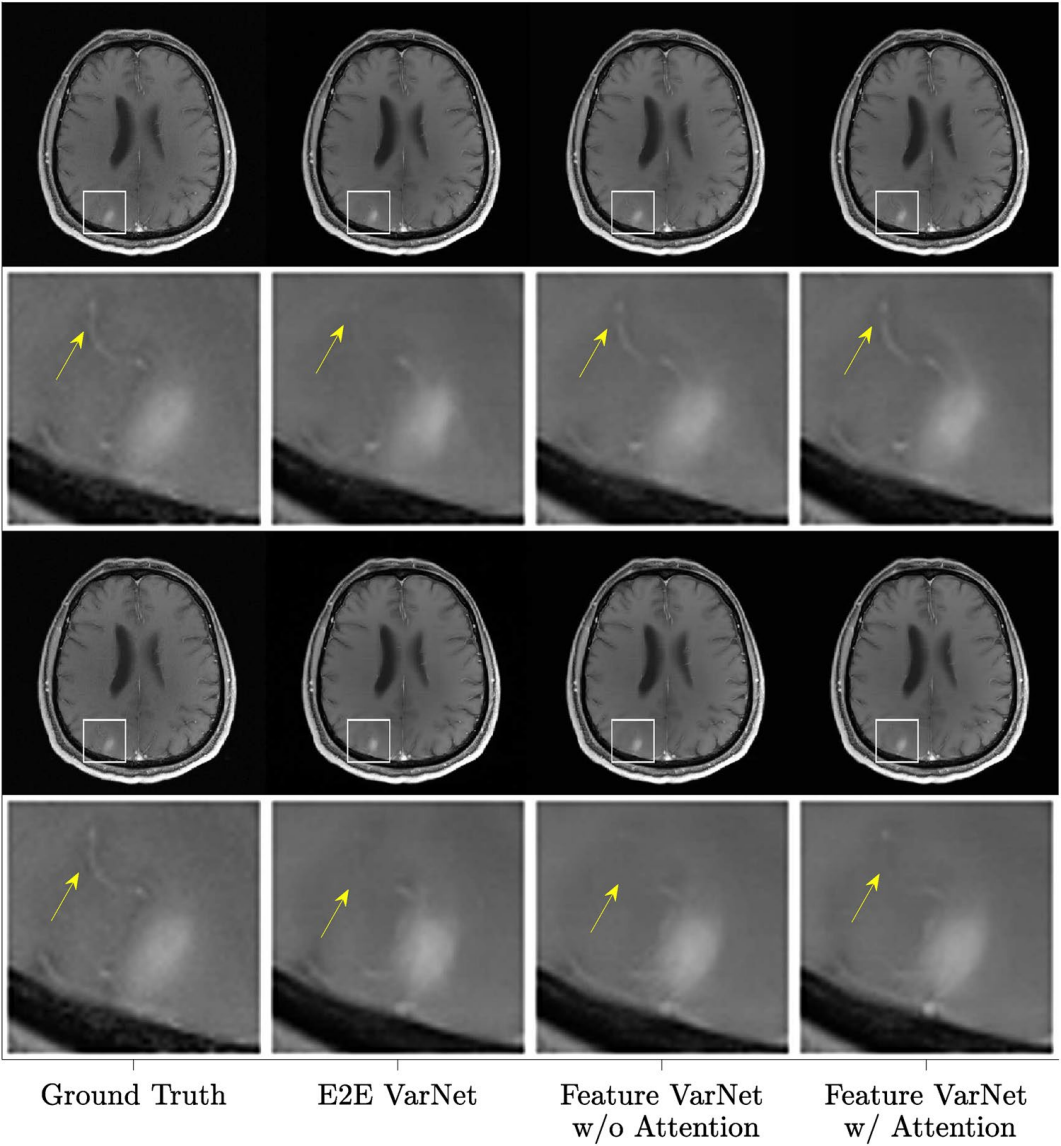
Comparison of representative image reconstructions at four-(top) and five-(bottom) fold accelerations using different VarNet architectures with matching train-test conditions. The fully-sampled ground truth reconstructions are shown on the left column. A zoomed section of interest, indicated by the white bounding box, is shown at even rows. The E2E VarNet blurs or misses the blood vessel next to the lesion in the zoomed area (yellow arrow) at $4\times$ and $5\times$ acceleration factors, respectively. The vessel is visible with the Feature VarNet w/o attention at $4\times$ acceleration, but it is missed at $5\times$. The Feature VarNet w/ attention reconstruction is able to preserve the vessel at $4\times$ acceleration and blur it at $5\times$.

Figure 4 shows a representative reconstruction, in which the E2E VarNet resulted in an artifact in the zoomed area (yellow arrow), which varied with the two different acceleration factors. In contrast, the Feature (with attention) VarNet and FI VarNet reconstructions did not exhibit this artifact. Additionally, at $4 \times$ acceleration, the E2E VarNet caused blurring of the blood vessel visible on the left side of the panel, while the blood vessel remained visible in both the Feature VarNet and the FI VarNet reconstructions (red arrows). At $5 \times$ acceleration, all models resulted in a similar smoothing on the vessel. These results suggest that both the the Feature VarNet and FI VarNet architectures could be more robust to acceleration artifacts than the the E2E VarNet architecture. Figure 5 presents $4\times$ and $5\times$ accelerated reconstructions for another representative case. The E2E VarNet blurs a blood vessel next to the lesion in the zoomed area (yellow arrow) at $4\times$ acceleration and misses it at $5 \times$. The vessel is visible with the Feature VarNet w/o attention at $4\times$ acceleration, but it is missed at $5\times$. The Feature VarNet w/ attention reconstruction is able to preserve the vessel at $4\times$ acceleration and blurs it at $5\times$.

### Model ablations

We trained a Feature VarNet model without attention (12 cascades) and with distinct encoders and decoders at each cascade. We again used single convolutional layers to represent them, but this time their weights were not shared between different cascades. The network was tested on the fastMRI brain test dataset and yielded a slight enhancement in both SSIM and PSNR (0.9591 and 41.41) compared to using consistent encoders and decoders (0.9589 and 41.39). This ablation in the Feature VarNet introduced 12 (as many as the gradient descent iterations) unique feature spaces, which provided increased flexibility during the training.

We also explored the performance of an image-feature (IF) VarNet with 6+6 cascades and four-fold accelerated brain MRI reconstructions. This model achieved SSIM and PSNR scores of 0.9596 and 41.35, respectively, on the fastMRI test dataset. The SSIM was equal to the one obtained with the FI VarNet of the same size, while the PSNR was lower by 0.1.

### Leaderboard comparison

We evaluated our FI VarNet model, which yielded the highest performance in this study, against the leading models on the fastMRI public leaderboard^18,67^, including the pretrained E2E VarNet model^16^. To ensure a fair comparison, we tested our model on the same leaderboard test dataset used by the other models (Datasets). To enhance our model’s performance, we increased the number of cascades from 6 to 12 in both its feature and image sub-network components. This does not compromise the fairness of the comparison, since the memory and operations complexity vary across all networks submitted in the public leaderboard^67^. Table 2 includes the comparison results for four-fold and eight-fold accelerations. For four-fold accelerations our FI VarNet model outperformed the DIRCN model (which is based on densely interconnected networks and the E2E VarNet architecture) by 0.0006 and 0.2 in terms of SSIM and PSNR, respectively. For eight-fold accelerations, FI VarNet was marginally outperformed by DIRCN in SSIM by 0.0002, but its PSNR was larger by 0.02. These results position our model in second place and third place on the leaderboard, just below the AIRS-Net, which is a closed-source model from AIRS Medical (Seoul, South Korea). Two screenshots of the public leaderboard are provided in the supplementary information for reference.

**Table 2:** Average SSIM and PSNR on the leaderboard test dataset for the top six models in the fastMRI public leaderboard.

Ranking	Model	SSIM	PSNR
4× acceleration			
$1^\mathrm{st}$	AIRS-Net	0.9632	42.1
$2^\mathrm{nd}$	FI VarNet	0.9607	41.5
$3^\mathrm{rd}$	DIRCN	0.9601	41.3
$4^\mathrm{th}$	E2E VarNet	0.9591	41.1
$5^\mathrm{th}$	dd	0.9591	41.1
$6^\mathrm{th}$	IR_FRestormerF11	0.9587	41.0
8× acceleration			
$1^\mathrm{st}$	AIRS-Net	0.9511	39.7
$2^\mathrm{nd}$	DIRCN	0.9455	38.6
$3^\mathrm{rd}$	FI VarNet	0.9453	38.6
$4^\mathrm{th}$	IR_FRestormerF72	0.9427	38.0
$5^\mathrm{th}$	E2E VarNet	0.9426	38.0
$6^\mathrm{th}$	dd	0.9426	38.0

The FI VarNet surpassed DIRCN at 4$\times$ (top) accelerated reconstructions and ranked second. For 8$\times$ (bottom) accelerated reconstructions FI VarNet was marginally outperformed by DIRCN and ranked third.

### Clinical evaluation

Table 3 includes the preference for each reader in terms of quality of the reconstructed images. On average, the readers preferred the FI VarNet in $\sim 62 \%$ of the 20 cases. For $7\%$ of the cases (2 cases for Reader A and 2 cases Reader B), the readers rated no major differences in the overall quality. Overall, the differences between the readers was non-significant based on the Wilcoxon signed-rank test^69^. The p-values were 0.1, 0.16, and 0.39, for the findings of reader A vs. C, reader B vs. C, and reader A vs. B, respectively.

**Table 3:** Comparison of the three readers’ scores in terms of image quality preference (as number of cases), artifacts, sharpness and contrast-to-noise ratio (CNR) for four-fold accelerated reconstructions with the FI VarNet and E2E VarNet models for four-fold accelerated reconstructions.

Network	Attribute	Reader A	Reader B	Reader C
FI VarNet	Preference	$\mathbf {13/20}$	$\mathbf {12/20}$	$\mathbf {12/20}$
	Artifacts	$1.85 \pm 0.36$	$1.05 \pm 0.22$	$1.75 \pm 0.44$
	Sharpness	$\mathbf {1.45 \pm 0.51}$	$\mathbf {1.30 \pm 0.47}$	$2.00 \pm 0.00$
	CNR	$1.20 \pm 0.41$	$\mathbf {1.15 \pm 0.36}$	$\mathbf {1.05 \pm 0.22}$
E2E VarNet	Preference	5/20	6/20	8/20
	Artifacts	$\mathbf {1.80 \pm 0.41}$	$1.05 \pm 0.22$	$1.75 \pm 0.44$
	Sharpness	$1.75 \pm 0.44$	$1.40 \pm 0.50$	$2.00 \pm 0.00$
	CNR	$\mathbf {1.15 \pm 0.36}$	$1.35 \pm 0.48$	$1.20 \pm 0.41$

Bold values indicate the best performing model.

Table 3 also reports the comparison among the three readers in terms of reconstruction artifacts, image sharpness and CNR. Reader A gave similar scores to the FI VarNet and E2E VarNet in terms of reconstruction artifacts and CNR, while scored the FI VarNet higher than the E2E model in terms of image sharpness. Reader B returned the same scores for artifacts for both models, and higher sharpness and CNR scores for the FI VarNet. Reader C returned the same scores for artifacts and sharpness for both networks and a higher CNR score for the FI VarNet.

**Figure 6:**
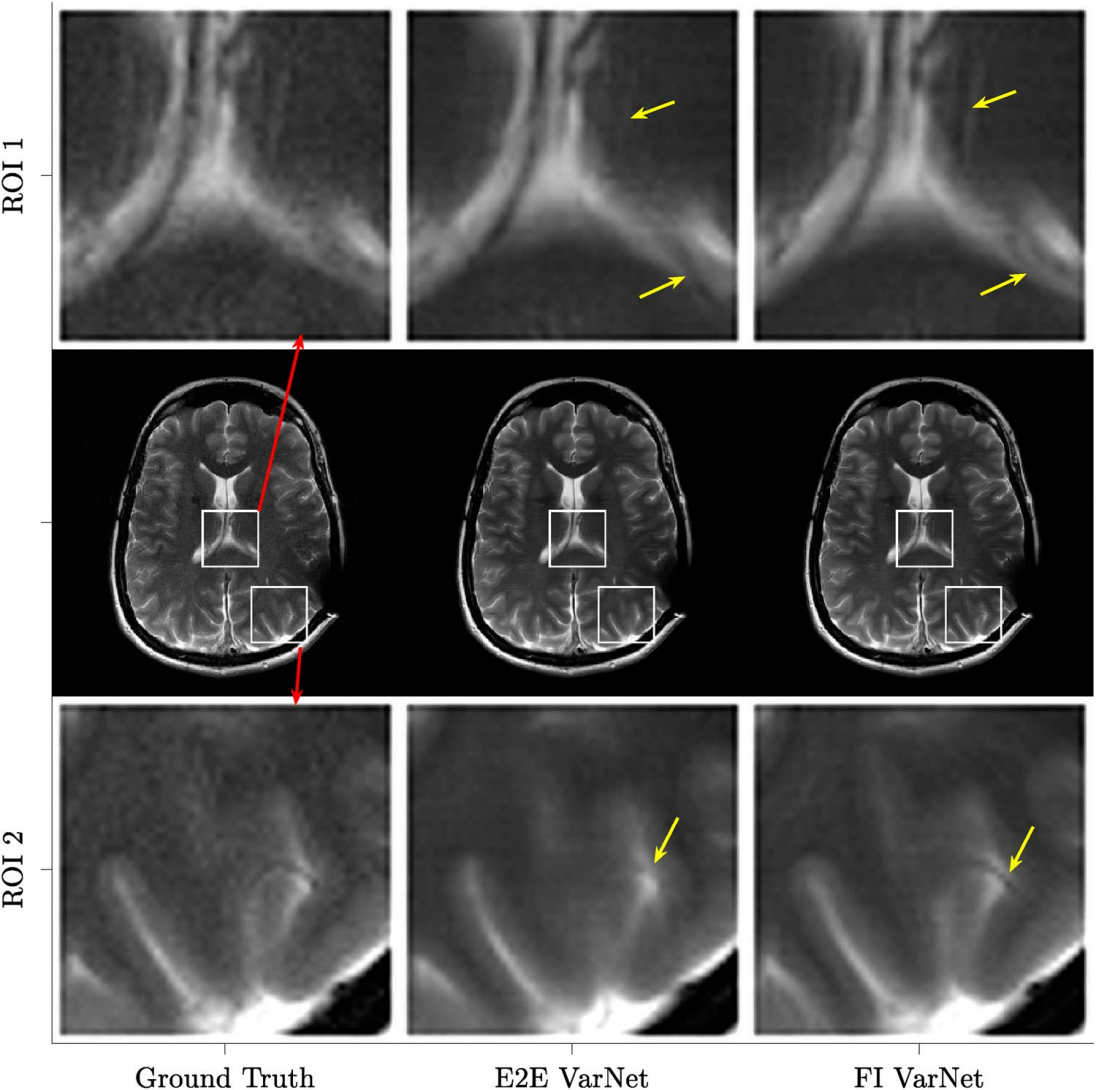
Comparison against the fully-sampled ground-truth of image reconstructions obtained using the FI VarNet and E2E VarNet models for four-fold undersampling. The data are from a representative T2-weighted brain MRI from the clinical dataset. The inset panels in the first and third row correspond to two zoomed ROIs indicated by the white bounding boxes in the images. The ground truth image was obtained as the root sum of squares of the individual coil images, using the fully sampled k-space. In ROI 1 the choroid plexus exhibits an artifact and the detail in the thalamus is blurred with the E2E VarNet reconstruction, as indicated by the two yellow arrows. The FI VarNet preserves the anatomy of the ground-truth. In ROI 2, the E2E VarNet misses the blood vessel pointed by the yellow arrow in the back of the brain, which instead remains visible in the FI VarNet reconstruction.

Figure 6 compares a representative T2-weighted brain image from the clinical dataset at four-fold acceleration, reconstructed using the FI VarNet and the E2E VarNet. Zoomed regions of interest (ROI) are shown to qualitatively compare the performance of the two models in capturing intricate details. In ROI 1, the E2E VarNet reconstruction exhibits a blurred representation of the thalamus and an artifact in the choroid plexus, which is highlighted by two yellow arrows. On the other hand, the FI VarNet model successfully preserves the anatomical features present in the ground truth. In ROI 2, the E2E VarNet reconstruction fails to capture a blood vessel indicated by the yellow arrow. The FI VarNet instead accurately retains the blood vessel.

### Performance on the knee

**Figure 7:**
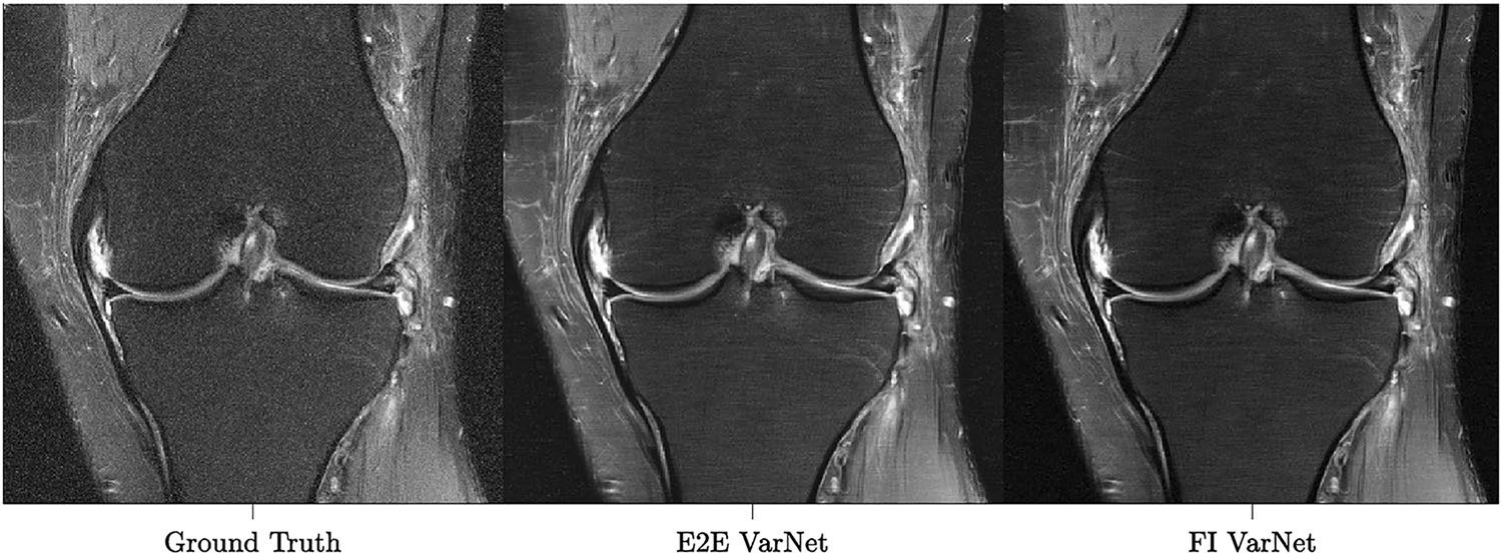
Comparison of representative knee image reconstructions at four-fold accelerations using the E2E VarNet (12 cascades) and the FI VarNet (12+12 cascades). The fully-sampled ground truth reconstruction is shown on the left column. The E2E VarNet exhibits higher levels of noise in the reconstruction. The noise is reduced in the FI VarNet reconstructions.

We trained and evaluated our best FI VarNet model (12 feature and 12 image cascades) on the knee fastMRI dataset^18,67^, and compared the reconstructions against the E2E VarNet model^16^. Table 1 (bottom) shows a comparison of the average SSIM, PSNR, and NMSE between the FI VarNet and E2E VarNet for the knee test dataset. The FI VarNet model outperformed the E2E model by 0.0049, 0.15, and 0.00013 in terms of SSIM, PSNR, and NMSE, respectively. These results show good generalizability of FI VarNet to other body regions. Figure 7 qualitatively shows that the FI VarNet can reduce noise compared to the E2E VarNet for the case of a four-fold under-sampled knee image reconstruction obtained with a fat-saturation sequence.

## Discussion

The E2E VarNet has established itself as a formidable tool in image reconstruction, standing out as one of the top open-source models in the fastMRI leaderboard^19,20^. Therefore, our aim in this work was not to replace the model but to refine it, introducing subtle architectural modifications that neither escalate its memory demands nor prolong training time and inference time. Our proposed Feature VarNet and FI VarNet architectures outperformed the E2E VarNet architecture in terms of image quality metrics such as SSIM, PSNR, and NMSE for four-, five-, and eight-fold undersampling rates. Unlike other feature-representation based networks^21,70^ our Feature VarNet’s feature space, defined by a single convolution layer, facilitates the incorporation of the block-wise attention transformer at a pixel resolution-level feature-space representation of the image. We note that a direct application of attention to raw image pixels would be suboptimal^50^. This single-layer convolutional encoding provides a direct representation of the image in feature space, with aliasing artifacts approximately mirroring their locations in the image domain (Supplementary Fig. S3). As a result, attention allows the network to attend directly to the aliasing artifacts in the phase-encoding direction due to the Cartesian undersampling (a key factor for its improved performance) and performs better denoising of the reconstruction through the network’s training Fig. 2). Finally, the marginal improvement observed in the ablated Feature VarNet, where encoders and decoders with non-shared weights are used, suggests that different architectures (other CNNs or transformers^71^) could be employed for the encoders and decoders to potentially enhance performance further.

Similar to KIKI-Net-based models^40^, the cross-domain architecture of the FI VarNet brings together the advantages of image (a purely comprehensive CNN model) and feature space (a CNN model augmented with attention mechanisms) networks, improving the overall reconstruction performance. FI VarNet outperformed other cross-domain learning-based networks that do not rely on unrolled optimization schemes. For example, CDF-Net^72^ had reported 0.9003 SSIM and 36.77 PSNR scores for 4$\times$ accelerated knee image reconstructions, whereas FI VarNet achieved 0.9236 SSIM and 40.08 PSNR when trained on the same dataset (We note that FI VarNet was tested on the entire fastMRI knee validation dataset, while CDF-Net was tested on only half of such dataset). The histogram in Fig. 2 (middle, right) and the average values per contrast in Fig. 3 shows high consistency of these results across SSIM and PSNR. Although the average performance improvements are not large, the representative example in Fig. 4 and 5 demonstrates that both the Feature (with attention) and FI VarNet architectures are more robust to acceleration artifacts than the E2E VarNet architecture.

Our FI VarNet model reached the second place (4$\times$) and third place (8$\times$) on the fastMRI public leaderboard, behind the closed-source AIRS-Net, which performs additional data standardization methods and a multi-slice training process^73^. Such data standardization methods appear to be key to achieve clinically good reconstructions for the entirety of the fastMRI dataset at higher acceleration factors and are left for future work^64^. Our comparison with the leaderboard models demonstrates the effectiveness of FI VarNet in MRI image reconstruction tasks and its potential for further development. While the increase in SSIM and PSNR achieved by our models was quantitatively small (Table 2), it nevertheless resulted in improved image quality and clinical scores (Table 3). This is due to the fact that these metrics can correlate poorly with the radiologist’s evaluations. In fact, the appearance of subtle pathologies could be substantially altered in the MR images without a major change in SSIM, therefore small changes in SSIM could be significant for pathology detection if associated with localized improvements in image quality^19,67,74,75^.

Our best model, the FI VarNet with $12+12$ cascades, outperformed the pretrained E2E VarNet^16^ according to three expert neuroradiologists. The results of the clinical evaluation were not statistically significant, which was expected due to the small number of cases (20). Minor differences in scoring were anticipated due to the binary structure of the employed Likert scales, the different years of experience among the readers, and the fact that the reconstruction quality of both networks was clinically acceptable for a four-fold acceleration factor. The FI VarNet excelled in preserving anatomical details, including small blood vessels, whereas the E2E VarNet discarded or blurred them in a few cases (Fig. 6). These findings suggest that the FI VarNet could enable reconstructions with diagnostic quality at five-fold or six-fold accelerations in cases where the E2E VarNet falls short^64^.

Our FI VarNet model also outperformed the E2E VarNet model in four-fold accelerated knee reconstructions. The achieved 0.0049 SSIM improvement shows that FI VarNet can effectively handle different anatomies using a small training dataset, which underscore its potential to learn accelerated image reconstruction using datasets with limited number of cases^76^.

The Feature and FI VarNet architectures are capable of accommodating Cartesian sampling patterns. In random or learned^77^ undersampling patterns, special care must be taken into account for the attention layers, as we designed them to identify the location of the aliasing artifacts due only to Cartesian undersampling. For non-Cartesian sampling, our method, as most of the unrolled optimization networks, would be slow, since the FFT through the network must be replaced with the slower non-uniform FFT^78^.

We also explored several alternative approaches that yielded either comparable or suboptimal results compared to the final models reported in this manuscript. For example, in the Feature VarNet, we attempted to improve the attention mechanism by incorporating two or three sequential attention layers. However, this was challenging during training due to the instability of the attention gradients. For this reason, future work will focus on alternative attention frameworks^79^ to improve stability during training. For the FI VarNet, we experimented with an image-feature representation with six cascades for each space, a feature-image-feature-image representation with four cascades for each space, and a feature-image-k-space representation with six cascades for each space. However, we observed that these representations led to degraded or similar reconstructions compared to the feature-image model with six or twelve cascades per space. Although these alternative approaches did not yield the desired improvements, they provided valuable insights into the behavior of the VarNet models and highlight the potential for further optimization, for example, by changing the network layers used for feature encoding and decoding to more complex architectures.

## Conclusion

We introduced three architectural modifications to the E2E VarNet model, namely feature-space training, block-wise attention layers based on the spatial position of the aliasing artifacts, and cross-domain learning between a CNN and a CNN augmented with attention. We have demonstrated the advantages of integrating these changes into the E2E VarNet model, showing improved reconstruction performance both quantitatively and qualitatively. The proposed approaches could enable clinically acceptable reconstructions at higher acceleration factors than currently possible.

### Supplementary Information

Supplementary Figures.

## Data Availability

The datasets used in the current study were obtained from the fastMRI public database fastmri.med.nyu.edu. The reconstructed MR images with the proposed neural networks used in the current study are available from https://rb.gy/vlfa4b. The PyTorch code for our models is available at https://github.com/facebookresearch/fastMRI.

## References

[1] Sodickson DK, Manning WJ (1997). Simultaneous acquisition of spatial harmonics (SMASH): Fast imaging with radiofrequency coil arrays. Magn. Reson. Med..

[2] Pruessmann KP, Weiger M, Scheidegger MB, Boesiger P (1999). SENSE: Sensitivity encoding for fast MRI. Magn. Reson. Med..

[3] Griswold MA (2002). Generalized autocalibrating partially parallel acquisitions (GRAPPA). Magn. Reson. Med..

[4] Lustig M, Donoho DL, Santos JM, Pauly JM (2008). Compressed sensing MRI. IEEE Signal Process. Mag..

[5] Raj A (2007). Bayesian parallel imaging with edge-preserving priors. Magn. Reson. Med..

[6] Uecker M, Hohage T, Block KT, Frahm J (2008). Image reconstruction by regularized nonlinear inversion-joint estimation of coil sensitivities and image content. Magn. Reson. Med..

[7] Knoll F, Bredies K, Pock T, Stollberger R (2011). Second order total generalized variation (TGV) for MRI. Magn. Reson. Med..

[8] Knoll F, Clason C, Bredies K, Uecker M, Stollberger R (2012). Parallel imaging with nonlinear reconstruction using variational penalties. Magn. Reson. Med..

[9] Uecker M (2014). ESPIRiT-an eigenvalue approach to autocalibrating parallel MRI: Where SENSE meets GRAPPA. Magn. Reson. Med..

[10] Muckley MJ, Noll DC, Fessler JA (2014). Fast parallel MR image reconstruction via B1-based, adaptive restart, iterative soft thresholding algorithms (BARISTA). IEEE Trans. Med. Imaging.

[11] Shin PJ (2014). Calibrationless parallel imaging reconstruction based on structured low-rank matrix completion. Magn. Reson. Med..

[12] Holme HCM (2019). ENLIVE: An efficient nonlinear method for calibrationless and robust parallel imaging. Sci. Rep..

[13] Hammernik K (2018). Learning a variational network for reconstruction of accelerated MRI data. Magn. Reson. Med..

[14] Knoll F (2020). Deep-learning methods for parallel magnetic resonance imaging reconstruction: A survey of the current approaches, trends, and issues. IEEE Signal Process. Mag..

[15] Roth S, Black MJ (2009). Fields of experts. Int. J. Comput. Vision.

[16] Sriram, A. *et al.* End-to-end variational networks for accelerated MRI reconstruction. In *Medical Image Computing and Computer Assisted Intervention–MICCAI 2020: 23rd International Conference, Lima, Peru, October 4–8, 2020, Proceedings, Part II 23*, 64–73 (Springer, 2020).

[17] Ronneberger, O., Fischer, P. & Brox, T. U-net: Convolutional networks for biomedical image segmentation. In *Medical Image Computing and Computer-Assisted Intervention–MICCAI 2015: 18th International Conference, Munich, Germany, October 5-9, 2015, Proceedings, Part III 18* 234–241 (Springer, 2015).

[18] Zbontar, J. *et al.* fastMRI: An open dataset and benchmarks for accelerated MRI. arXiv:1811.08839 (2018).

[19] Knoll F (2020). Advancing machine learning for MR image reconstruction with an open competition: Overview of the 2019 fastMRI challenge. Magn. Reson. Med..

[20] Muckley, M. J. *et al.**State-of-the-art Machine Learning MRI Reconstruction in 2020: Results of the Second fastMRI Challenge, vol. 2* 7. arXiv:2012.06318 (2020).

[21] Zhu B, Liu JZ, Cauley SF, Rosen BR, Rosen MS (2018). Image reconstruction by domain-transform manifold learning. Nature.

[22] Sandino CM, Lai P, Vasanawala SS, Cheng JY (2021). Accelerating cardiac cine MRI using a deep learning-based ESPIRiT reconstruction. Magn. Reson. Med..

[23] Hammernik K (2023). Physics-driven deep learning for computational magnetic resonance imaging: Combining physics and machine learning for improved medical imaging. IEEE Signal Process. Mag..

[24] Ottesen JA, Caan MW, Groote IR, Bjørnerud A (2022). A densely interconnected network for deep learning accelerated MRI. Magn. Reson. Mater. Phys. Biol. Med..

[25] Schlemper, J., Caballero, J., Hajnal, J. V., Price, A. & Rueckert, D. A deep cascade of convolutional neural networks for MR image reconstruction. In *Information Processing in Medical Imaging: 25th International Conference, IPMI 2017, Boone, NC, USA, June 25-30, 2017, Proceedings 25* 647–658 (Springer, 2017).

[26] Hosseini SAH, Yaman B, Moeller S, Hong M, Akçakaya M (2020). Dense recurrent neural networks for accelerated MRI: History-cognizant unrolling of optimization algorithms. IEEE J. Sel. Top. Signal Process..

[27] Yiasemis, G., Sonke, J.-J., Sánchez, C. & Teuwen, J. Recurrent variational network: A deep learning inverse problem Solver applied to the task of accelerated MRI reconstruction. In *Proceedings of the IEEE/CVF conference on computer vision and pattern recognition* 732–741 (2022).

[28] Arvinte, M., Vishwanath, S., Tewfik, A. H. & Tamir, J. I. Deep J-Sense: Accelerated MRI reconstruction via unrolled alternating optimization. In *Medical Image Computing and Computer Assisted Intervention–MICCAI 2021: 24th International Conference, Strasbourg, France, September 27–October 1, 2021, Proceedings, Part VI 24* 350–360 (Springer, 2021). 10.1007/978-3-030-87231-1_34 PMC8767765 35059693

[29] Jun, Y., Shin, H., Eo, T. & Hwang, D. Joint deep model-based MR image and coil sensitivity reconstruction network (joint-ICNet) for fast MRI. In *Proceedings of the IEEE/CVF Conference on Computer Vision and Pattern Recognition* 5270–5279 (2021).

[30] Cheng J (2021). Learning data consistency and its application to dynamic MR imaging. IEEE Trans. Med. Imaging.

[31] Qin, C. *et al.* Complementary time-frequency domain networks for dynamic parallel MR image reconstruction. In *Magnetic Resonance in Medicine* 3274–3291 (2021). 10.1002/mrm.28917 34254355

[32] Küstner, T. *et al.* CINENet: Deep learning-based 3D cardiac CINE MRI reconstruction with multi-coil complex-valued 4D spatio-temporal convolutions. In *Scientific reports* 13710 (2020). 10.1038/s41598-020-70551-8 PMC7426830 32792507

[33] Huang W (2021). Deep low-rank plus sparse network for dynamic MR imaging. Med. Image Anal..

[34] Yaman B (2020). Self-supervised learning of physics-guided reconstruction neural networks without fully sampled reference data. Magn. Reson. Med..

[35] Yoo J (2021). Time-dependent deep image prior for dynamic MRI. IEEE Trans. Med. Imaging.

[36] Hu, C. *et al.* Self-supervised learning for mri reconstruction with a parallel network training framework. In *Medical Image Computing and Computer Assisted Intervention–MICCAI 2021: 24th International Conference, Strasbourg, France, September 27–October 1, 2021, Proceedings, Part VI 24* 382–391 (Springer, 2021).

[37] Yaman B (2022). Multi-mask self-supervised learning for physics-guided neural networks in highly accelerated magnetic resonance imaging. NMR Biomed..

[38] Aali, A., Arvinte, M., Kumar, S. & Tamir, J. I. *Solving Inverse Problems with Score-Based Generative Priors learned from Noisy Data*. arXiv:2305.01166 (2023).

[39] Cui, Z.-X. *et al.* Self-score: Self-supervised learning on score-based models for mri reconstruction. arXiv:2209.00835 (2022).

[40] Eo T (2018). KIKI-net: Cross-domain convolutional neural networks for reconstructing undersampled magnetic resonance images. Magn. Reson. Med..

[41] Peng, Z. A Deep residual sparse and cross domain reconstruction network for dynamic MR imaging. In *Proceedings of the 2020 9th International Conference on Computing and Pattern Recognition* 350–355 (2020).

[42] Wang B (2024). DCT-net: Dual-domain cross-fusion transformer network for MRI reconstruction. Magn. Resonan. Imaging.

[43] Liu X (2022). Dual-domain reconstruction network with V-Net and K-Net for fast MRI. Magn. Resonan. Med..

[44] Liu Y (2023). DIIK-Net: A full-resolution cross-domain deep interaction convolutional neural network for MR image reconstruction. Neurocomputing.

[45] Ramzi, Z. XPDNet for MRI reconstruction: An application to the 2020 fastMRI challenge. arXiv:2010.07290 (2020).

[46] Wang Z (2020). IKWI-net: A cross-domain convolutional neural network for undersampled magnetic resonance image reconstruction. Magn. Resonan. Imaging.

[47] Shimron E (2022). Implicit data crimes: Machine learning bias arising from misuse of public data. Proc. Natl. Acad. Sci..

[48] Guerquin-Kern M (2011). Realistic analytical phantoms for parallel magnetic resonance imaging. IEEE Trans. Med. Imaging.

[49] Darestani, M. *et al.* IR-FRestormer: Iterative refinement with fourier-based restormer for accelerated MRI reconstruction. In *Proceedings of the IEEE/CVF Winter Conference on Applications of Computer Vision* (2024).

[50] Dosovitskiy, A. *et al.* An image is worth 16x16 words: Transformers for image recognition at scale. arXiv:2010.11929 (2020).

[51] Vaswani A (2017). Attention is all you need. Adv. Neural Inf. Process. Syst..

[52] Giannakopoulos II, Johnson P, Lattanzi R, Muckley MJ (2023). Improving variational network based 2D MRI reconstruction via feature-space data consistency. Proc. ISMRM.

[53] Giannakopoulos, I. I., Johnson, P., Lattanzi, R. & Muckley, M. J. Improving variational network based 2D MRI reconstruction via feature-space data consistency. In *ISMRM Data Sampling & Image Reconstruction Workshop* 35 (2023).

[54] Candès EJ, Romberg J, Tao T (2006). Robust uncertainty principles: Exact signal reconstruction from highly incomplete frequency information. IEEE Trans. Inf. Theory.

[55] Hollingsworth KG (2015). Reducing acquisition time in clinical MRI by data undersampling and compressed sensing reconstruction. Phys. Med. Biol..

[56] Lefkimmiatis, S. Non-local color image denoising with convolutional neural networks. In *Proceedings of the IEEE Conference on Computer Vision and Pattern Recognition* 3587–3596 (2017). 10.1109/CVPR.2017.506 PMC6191179 30337799

[57] Aggarwal HK, Mani MP, Jacob M (2018). MoDL: Model-based deep learning architecture for inverse problems. IEEE Trans. Med. Imaging.

[58] Gilton D, Ongie G, Willett R (2021). Deep equilibrium architectures for inverse problems in imaging. IEEE Trans. Comput. Imaging.

[59] Korhonen, J. & You, J. Peak signal-to-noise ratio revisited: Is simple beautiful? In *2012 Fourth International Workshop on Quality of Multimedia Experience* 37–38 (IEEE, 2012).

[60] Wang, Z., Simoncelli, E. P. & Bovik, A. C. Multiscale structural similarity for image quality assessment. *The Thrity-Seventh Asilomar Conference on Signals, Systems & Computers*, **2**, 1398–1402 (IEEE, 2003).

[61] Wang S, Xiao T, Liu Q, Zheng H (2021). Deep learning for fast MR imaging: A review for learning reconstruction from incomplete k-space data. Biomed. Signal Process. Control.

[62] Ramzi Z, Ciuciu P, Starck J-L (2020). Benchmarking MRI reconstruction neural networks on large public datasets. Appl. Sci..

[63] Knoll F (2020). fastMRI: A publicly available raw k-space and DICOM dataset of knee images for accelerated MR image reconstruction using machine learning. Radiol. Artif. Intell..

[64] Radmanesh A (2022). Exploring the acceleration limits of deep learning variational network-based two-dimensional brain MRI. Radiol. Artif. Intell..

[65] Loshchilov, I. & Hutter, F. Decoupled weight decay regularization. arXiv:1711.05101 (2017).

[66] Hore, A. & Ziou, D. Image quality metrics: PSNR vs. SSIM. In *2010 20th International Conference on Pattern Recognition* 2366–2369 (IEEE, 2010).

[67] Muckley MJ (2021). Results of the 2020 fastMRI challenge for machine learning MR image reconstruction. IEEE Trans. Med. Imaging.

[68] Hsu, H. & Lachenbruch, P. A. Paired t test. In *Wiley StatsRef: statistics reference online* (2014).

[69] Woolson RF (2007). Wilcoxon signed-rank test. Wiley Encycl. Clin. Trials.

[70] Jiang, J. Latent-space Unfolding for MRI Reconstruction. In *Proceedings of the 31st ACM International Conference on Multimedia* 1294–1302 (2023).

[71] Zhai, X. *et al.* Scaling vision transformers. In *Proceedings of the IEEE/CVF Conference on Computer Vision and Pattern Recognition* 3274–3291 (2022).

[72] Nitski, O. Cdf-net: Cross-domain fusion network for accelerated mri reconstruction. In *International Conference on Medical Image Computing and Computer-Assisted Intervention* 421–430 (2020).

[73] Kim, S. Feature-level multi-domain learning with a standardization for multichannel MRI data*, *In* Medical Imaging Meets NeurIPS* (2020).

[74] Mason A (2019). Comparison of objective image quality metrics to expert radiologists’ scoring of diagnostic quality of MR images. IEEE Trans. Med. Imaging.

[75] Calivá, F., Cheng, K., Shah, R. & Pedoia, V. Adversarial robust training of deep learning MRI reconstruction models. arXiv:2011.00070 (2020).

[76] Tibrewala, R. *et al.* FastMRI prostate: A publicly available, biparametric MRI dataset to advance machine learning for prostate cancer imaging. arXiv:2304.09254 (2023). 10.1038/s41597-024-03252-w PMC11032332 38643291

[77] Zibetti MVW, Knoll F, Regatte RR (2022). Alternating learning approach for variational networks and undersampling pattern in parallel MRI applications. IEEE Trans. Comput. Imaging.

[78] Greengard L, Lee J-Y (2004). Accelerating the nonuniform fast Fourier transform. SIAM Rev..

[79] Wang, Q. *et al.* ECA-Net: Efficient channel attention for deep convolutional neural networks. In *Proceedings of the IEEE/CVF conference on computer vision and pattern recognition* 11534–11542 (2020).

