# Supplementary material for: Accelerated MRI reconstructions via variational network and feature domain learning
Source: Sci Rep. 2024 May 14;14:10991. doi: 10.1038/s41598-024-59705-0 (PMC11094153; doi:10.1038/s41598-024-59705-0)
Supplement: Supplementary file 1 — Supplementary Figures. [file 41598_2024_59705_MOESM1_ESM.pdf]

**Supplementary Figures** for the manuscript:

Accelerated MRI Reconstructions via Variational Network and Feature Domain Learning.

Ilias I. Giannakopoulos[1,*], Matthew J. Muckley[2], Jesi Kim[1], Matthew Breen[1] Patricia M. Johnson[1,3,4], Yvonne W. Lui[1,3,4], Riccardo Lattanzi[1,2,4]

[1]*The Bernard and Irene Schwartz Center for Biomedical Imaging, Department of Radiology, New York University Grossman School of Medicine, New York, NY, 10016, United States*,
[2]*Meta AI Research, New York, NY, 10003, United States*,
[3]*Center for Advanced Imaging Innovation and Research (CAI$^2$R), Department of Radiology, New York University Grossman School of Medicine, New York, NY, 10016, United States*.
[4]*Vilcek Institute of Graduate Biomedical Sciences, New York University Grossman School of Medicine, New York, NY, 10016, USA*.
[*]*ilias.giannakopoulos@nyulangone.org*.

| | Acceleration 4x | NMSE | SSIM | PSNR | NYU DATA ONLY |
|---|---|---|---|---|---|
| AIRS-Net 10/18/2020 | 4x | 0.0029 | 0.9632 | 42.1 | ✓ |
| DIRCN 9/26/2022 | 4x | 0.0035 | 0.9601 | 41.3 | ✓ |
| fastMRI Repo End-to-End VarNet 11/11/2020 | 4x | 0.0037 | 0.9591 | 41.1 | ✓ |
| dd 11/27/2020 | 4x | 0.0037 | 0.9591 | 41.1 | ✓ |
| IR_FRestormerF11 2/15/2023 | 4x | 0.0037 | 0.9589 | 41.0 | ✓ |

**Supplementary Figure S1.** Latest screenshot of the fastMRI public leaderboard before its shutdown in April 2023. The leaderboard ranks the performance of various models on the task of reconstructing brain MRI from $4\times$ undersampled k-space data. Our FI VarNet model achieved second place on the leaderboard, below the AIRS-Net model. The table displays the average SSIM, PSNR, and NMSE scores for the 5 top-performing models.

| | Acceleration 8x | NMSE | SSIM | PSNR | NYU DATA ONLY |
|---|---|---|---|---|---|
| AIRS-Net 10/18/2020 | 8x | 0.0052 | 0.9511 | 39.7 | ✓ |
| DIRCN 9/26/2022 | 8x | 0.0066 | 0.9455 | 38.6 | ✓ |
| IR_FRestormerF72 12/18/2022 | 8x | 0.0075 | 0.9427 | 38.0 | ✓ |
| fastMRI Repo End-to-End VarNet 11/11/2020 | 8x | 0.0075 | 0.9426 | 38.0 | ✓ |
| dd 11/27/2020 | 8x | 0.0075 | 0.9426 | 38.0 | ✓ |

**Supplementary Figure S2.** Latest screenshot of the fastMRI public leaderboard before its shutdown in April 2023. The leaderboard ranks the performance of various models on the task of reconstructing brain MRI from $8\times$ undersampled k-space data. Our FI VarNet model achieved third place on the leaderboard, below the DIRCN model. The table displays the average SSIM, PSNR, and NMSE scores for the 5 top-performing models.

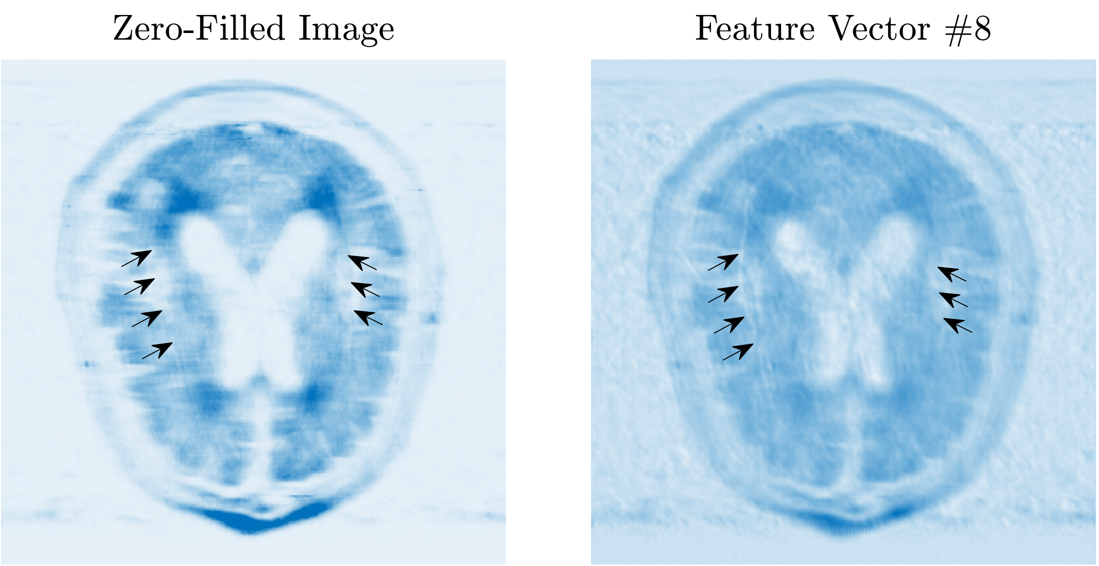


**Supplementary Figure S3.** (left) Zero-filled brain image acquired with four times Cartesian undersampling. (right) Representative feature vector after the initial encoding of the corresponding k-space during testing with the Feature VarNet (w/ attention). Both the image and the feature vector preserve the aliasing artifacts at roughly the same locations (for example, the ones pointed with the black arrows).
